# Hypoxia Induces EMT in Low and Highly Aggressive Pancreatic Tumor Cells but Only Cells with Cancer Stem Cell Characteristics Acquire Pronounced Migratory Potential

**DOI:** 10.1371/journal.pone.0046391

**Published:** 2012-09-26

**Authors:** Alexei V. Salnikov, Li Liu, Mitja Platen, Jury Gladkich, Olga Salnikova, Eduard Ryschich, Jürgen Mattern, Gerhard Moldenhauer, Jens Werner, Peter Schemmer, Markus W. Büchler, Ingrid Herr

**Affiliations:** 1 Molecular OncoSurgery Group, Department of General Surgery, University of Heidelberg and German Cancer Research Center, Heidelberg, Germany; 2 Department of Translational Immunology, German Cancer Research Center and National Center for Tumor Diseases, Heidelberg, Germany; 3 Department of General Surgery, University of Heidelberg, Heidelberg, Germany; Wayne State University School of Medicine, United States of America

## Abstract

Tumor hypoxia induces epithelial-mesenchymal transition (EMT), which induces invasion and metastasis, and is linked to cancer stem cells (CSCs). Whether EMT generates CSCs *de novo*, enhances migration of existing CSCs or both is unclear. We examined patient tissue of pancreatic ductal adenocarcinoma (PDA) along with carcinomas of breast, lung, kidney, prostate and ovary. For *in vitro* studies, five established PDA cell lines classified as less (CSC^low^) and highly aggressive CSC-like cells (CSC^high^) were examined by single and double immunofluorescence microscopy, wound-, transwell-, and time-lapse microscopy. HIF-1α and Slug, as well as HIF-2α and CD133 were co-expressed pointing to a putative co-existence of hypoxia, EMT and CSCs *in vivo*. CSC^high^ cells exhibited high basal expression of the mesenchymal Vimentin protein but low or absent expression of the epithelial marker E-cadherin, with the opposite result in CSC^low^ cells. Hypoxia triggered altering of cell morphology from an epithelial to a mesenchymal phenotype, which was more pronounced in CSC^high^ cells. Concomitantly, E-cadherin expression was reduced and expression of Vimentin, Slug, Twist2 and Zeb1 enhanced. While hypoxia caused migration in all cell lines, velocity along with the percentage of migrating, polarized and pseudopodia-forming cells was significantly higher in CSC^high^ cells. These data indicate that hypoxia-induced EMT occurs in PDA and several other tumor entities. However although hypoxia-induced EMT signaling occurs in all tumor cell populations, only the stem-like cells acquire high migratory potential and thus may be responsible for invasion and metastasis.

## Introduction

Pancreatic ductal adenocarcinoma (PDA) is an aggressive malignancy characterized by an extensive local invasion, early systemic dissemination and marked resistance to chemo- and radiotherapy [1]. In addition, most PDA possess a pronounced hypoxic tumor-microenvironment [2]. Tumor hypoxia occurs when the consumption of oxygen exceeds its delivery by the vascular system [3]. This leads to induction of hypoxia-inducible transcription factors, e.g. HIF-1α and HIF-2α, which regulate the hypoxic response by induction of target genes like VEGF [4]. The oxygen pressure in solid tumors is generally lower than in the surrounding non-malignant tissues, and tumors exhibiting extensive hypoxia have been shown to be more aggressive than corresponding tumors that are better oxygenized [5], [6], [7]. This includes pancreatic cancer where high expression of the hypoxia marker HIF-1α in patient tissue has been demonstrated to be a predictor of poor clinical outcome [8]. In experimental studies, hypoxia predicts aggressive growth and spontaneous metastasis formation in pancreatic cancer xenografts [9]. Accordingly, relapsed tumors show a higher hypoxic fraction compared to the primary tumors [10], [11] suggesting a role of hypoxia in enrichment of cancer cells with stem cell characteristics (CSC).

The small CSC population is suggested to possess self-renewal potential and the ability to differentiate and thereby generating the heterogenous cell population of the originating tumor [12], [13], [14]. These findings have been also demonstrated for pancreatic cancer [15], [16]. In addition CSC are proposed to mediate uncontrolled growth, therapy resistance, invasion and metastasis [17]. However, whether CSCs are truly the only cells with *de facto* tumorigenic potential remains controversial [18], [19], [20], [21]. Recent reports indicate that the emergence of CSCs occurs in part as a result of epithelial-mesenchymal-transition (EMT) [21]. Therefore the question arises whether **EMT affects the CSC population only or also the more differentiated progenitors**

EMT is an evolutionarily conserved development process during that cells lose epithelial characteristics and gain mesenchymal properties [22]. This is accompanied by the dissolution of cell-cell junctions and loss of apico-basolateral polarity, resulting in the formation of migratory mesenchymal cells with invasive properties [21]. Therefore, EMT is implicated in tumor progression and metastasis [23]. EMT-inducers, such as transforming growth factor-β (TGF-β) or hypoxia, trigger changes in gene expression by complex signaling pathways. A basic mechanism involved in progression of EMT is upregulation of the mesenchymal marker Vimentin and downregulation of the epithelial marker E-cadherin - the main transmembrane adhesion molecule responsible for cell-to-cell interactions and tissue organization in epithelial cells [24]. E-cadherin is transcriptionally repressed by Twist, Snail, Slug and Zeb proteins. Reduced E-cadherin expression causes adherens junction breakdown, and along with other signaling events promotes robust gene expression changes [21]. The loss of polarity and gain of motile characteristics of mesenchymal cells during embryonic development has prompted comparisons with metastatic cancer cells during malignant progression [25]. Notably, recent data demonstrate that EMT is indeed involved in generating cells with properties of stem cells as shown in cancer of the breast [25], [26], colorectum [27] and pancreas [28], [29].

According to the CSC hypothesis solely the CSC population is responsible for early systemic dissemination and metastasis formation. This implies that hypoxia-induced EMT either affects CSCs only or activates more differentiated progenitors to stem-like cells or both together. Since this issue is not examined so far, we addressed this question. By focusing to pancreatic cancer we found co-expression of hypoxia-, EMT and CSC markers in patient-derived tissue. By the use of established cell lines with high or low stem cells characteristics (CSC^high^ or CSC^low^) we induced hypoxia by a gas mixture of low oxygen. This led to changes in cell morphology resulting in a more fibroblastoid-phenotype and EMT-related protein expression in both tumor cell populations. However the more aggressive cells had a higher basal EMT-signature and this was associated with faster and higher hypoxia-mediated induction of migratory activity. Our findings may have implication for several tumor entities, since we found expression of the hypoxia marker CA IX and of the EMT marker Twist2 not only in PDA but also in patient-derived cancer tissue of breast, kidney, prostate, lung and ovary.

## Materials and Methods

### Tumor cell lines

BxPc-3, Capan-2, MIA-PaCa2, AsPC-1 and Capan-1 PDA cell lines were obtained from the American Type Culture Collection (Manassas, VA, USA) and authenticated throughout the culture by the typical morphology. Mycoplasma negative cultures were ensured by weekly tests. Cells were cultured in DMEM (PAA, Pasching, Austria) supplemented with 10% heat-inactivated FCS (Sigma-Aldrich, St. Louis, MO, USA) and 25 mmol/L HEPES (PAA, Pasching, Austria).

### Ethics Statement + Tumor tissue samples

Patient-derived tumor tissue from pancreatic, breast, renal, lung, prostate and ovarian cancer was obtained under the approval of the ethical committee of the University of Heidelberg. The tissue was analyzed anonymously and is derived from a 30-year-old tissue bank. Therefore a patient consent form is not applicable. Diagnoses were established by conventional clinical and histological criteria according to the World Health Organization (WHO). All clinical investigation have been conducted according to the principles expressed in the Declaration of Helsinki.

### In vitro hypoxia model

For induction of hypoxia 80% confluent cells were put in a hypoxia chamber (self-made), which was flushed by a gas mixture of 1% O_2_, 5% CO_2_, 94% N_2_ (Grandpair, Heidelberg, Germany) for about 4 min. Cells were incubated in the hypoxic environment for 24, 48 or 72 h at 37°C. The chamber was refilled with the gas mixture after 24 h to ensure constant gas concentrations.

### Immunohistochemistry and immunocytochemistry

Immunohistochemistry on 6 µm frozen or paraffin-embedded tissue sections was performed as described previously [30]. Antibodies used for immunohistochemistry were rabbit polyclonal anti-human CA IX (Santa Cruz Biotechnology) and mouse mAbs anti-human Twist2 and Vimentin (Abcam, Cambridge, UK). For double immunocytofluorescence stainings mouse mAbs directed towards HIF-1α (R&D Systems, Abingdon, UK), HIF-2α (Novus Biologicals, Cambridge, United Kingdom), and rabbit polyclonal antibodies against human Slug, CD133 (Abcam, Cambridge, United Kingdom), E-cadherin (Cell Signaling, Danvers, MA, USA) and Zeb1 (Santa Cruz Biotechnology, Heidelberg, Germany). Secondary antibodies were goat anti-mouse IgG conjugated to Alexa 488 or goat anti-rabbit IgG Alexa 594 (Invitrogen, Karlsruhe, Germany).

### Wound healing assay

Tumor cells (6×10^5^) were seeded in 6-well plates and grown to confluence overnight. Twenty-four hours after incubation under hypoxic or normoxic conditions a line was scratched within the confluent cell layer using the fine end of a 10 µl pipette tip (time 0). Images of migrating cells were sequentially taken during closure of the wounded region.

### Time-lapse video microscopy

BxPc-3 or AsPC-1 cells (5×10^5^) exposed to hypoxia or normoxia for 48 h were mixed with a collagen type I solution. A POCmini microscope chamber (LaCon GbR, Ulm, Germany) was filled with tumor cells in collagen solution and the gel was polymerized at 37°C in a CO_2_ incubator for 1.5 hours. The POCmini chamber with the polymerized three-dimensional (3D) collagen gel was then placed on a heating plate (36.6°C, LaCon, Ulm, Germany) under the microscope (Leitz, Wetzlar, Germany). Cells in 3D collagen gels were focused under 50*x* magnification. Images were recorded using a Kappa digital videocamera (DX2, Kappa GmbH, Gleichen, Germany) and were taken consequently with a 15 minutes interval during a period of 24 hours. Sequential TIFF images were transferred into the AVI-video format using Animation Shop software. The analyses of cell movements were performed with CapImage 8.4 software (Dr. Zeintl Biomedical Engineering, Heidelberg, Germany). Migration of single cells was analyzed under 2∶1 zoom using a “frame to frame” method. Percentage of migrating cells, type of movement and velocity were evaluated.

### Transwell migration assay

To analyze cell invasive potential we used a standard transwell assay described elsewhere. Transwell polycarbonate filter (8 μm pore size) (Corning Inc, Lowell, MA) were used. Hypoxia pre-treated (48 h) or normoxia-treated control cells were seeded at a concentration of 10^5^ cells per cm^2^ of 24-well plates. Afterwards the cells were exposed to hypoxia (Migration measured under Hypoxia) or normoxia (Migration measured under Hypoxia) for additional 48 h and the number of transmigrated cells was counted. The percentage of transmigrated cells was normalized to the percentage of cell vitality evaluated by an MTT assay at the endpoint of the experiment.

### Statistical analysis

Quantitative data are presented as the mean ± SD. Data were analyzed using the Student's *t* test for statistical significance. *P*<0.05 was considered statistically significant.

## Results

### Expression of hypoxia-, EMT- and CSC-markers in pancreatic cancer tissue

**Figure 1:**
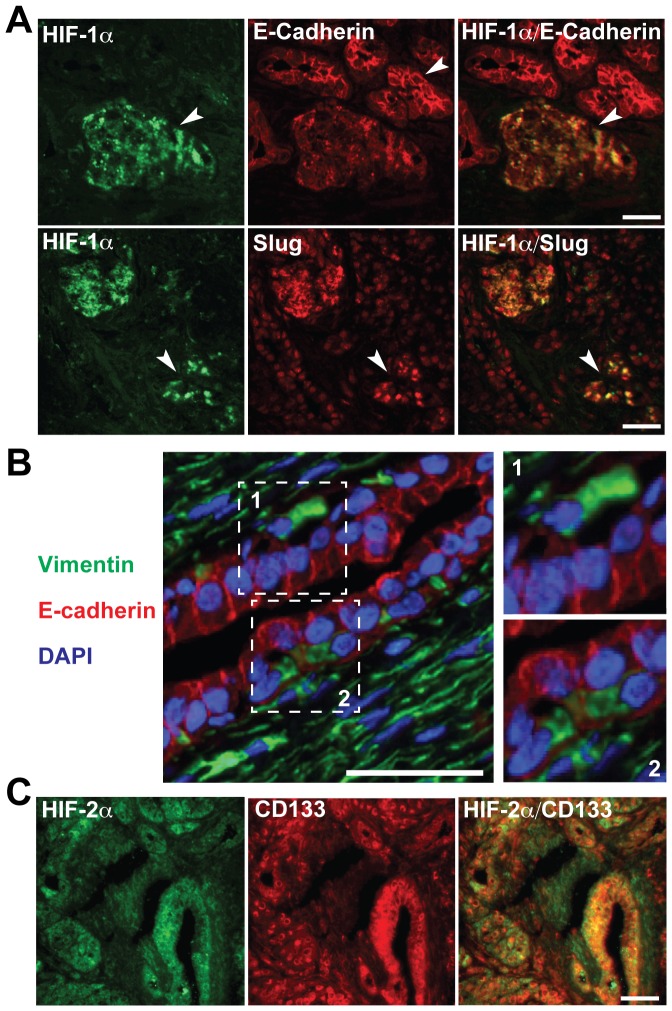
Co-expression of hypoxia, EMT and CSC markers in pancreatic cancer tissues. (**A**) Double immunofluorescence stainings of tumor samples from patients with pancreatic cancer. Nuclear expression of HIF-1α (green, arrow) and membrane expression of E-cadherin (red, arrow) or Slug (red, arrow) is shown. Yellow color on merged images indicates co-expression of E-cadherin or Slug in HIF-1α-positive cells. (**B**) Membrane expression of E-cadherin (red), cytoplasmic expression of Vimentin (green) along with Dapi-staining of nuclei (blue). White squares indicate Vimentin-positive cells within the tumor mass that are negative for E-cadherin or *vice versa*. Bar: 100 μm. Twofold magnifications of the areas surrounded by white squares are shown on the left. (**C**) Nuclear expression of HIF-2α and membrane expression of CD133 is shown as single staining and as merged staining in which the yellow color indicates double-positivity.

To study co-expression of hypoxia- and EMT-markers we performed double immunofluorescence staining of patient-derived frozen tissue samples of pancreatic ductal adenocarcinoma. In HIF-1α positive regions E-cadherin was down-regulated and Slug up-regulated revealing tumor-hypoxia-induced EMT (Fig. 1A). In some tumor areas, cells with either Vimentin or E-cadherin expression were observed in close proximity (Fig. 1B). This indicates that surrounding stromal cells are positive for Vimentin and negative for E-cadherin. Most interestingly, hypoxic regions positive for the hypoxia marker HIF-2α showed co-staining with CD133, suggesting that tumor hypoxia is associated with expression of CSC markers (Fig. 1C). These data confirm that hypoxia-driven EMT occurs in tumor tissue of patients with pancreatic cancer and CSC-positive tumor cells are present in hypoxic tumor microenvironments.

### Hypoxia induces HIF-1α signaling and morphological changes in vitro

For more detailed evaluation of hypoxia-induced EMT and the influence to differentiated and CSC-like pancreatic cancer cells we used five established human cell lines of PDA. According to the degree of differentiation of the primary tumor, mutations in K-ras or p53, colony- and spheroid-forming capacity, ALDH activity, tumorigenicity in mice and expression of E-cadherin and Vimentin we classified these cell lines as CSC^high^ or CSC^low^ (Table 1). Hypoxia was induced by incubation of cells in a gas mixture of 1% O_2_, 5% CO_2_ and 94% N_2_. This resulted in fast up-regulation of HIF-

**Table 1:** Characterization of CSC marker characteristics in established human pancreatic ductal adenocarcinoma cell lines.

	More aggressive CSC^high^ cells			Less aggressive CSC^low^ cells		References
	MIA-PaCa2	AsPC-1	Capan-1	BxPc-3	Capan-2	
**ATCC No.**	CRL-1420	CRL-1682	HRB 79	CRL-1687	HTB-80	ATCC
**Source**	Primary tumor	Ascites	Liver Metastasis	Primary tumor	Primary tumor	ATCC
**Differentiation** **of primary tumor**	Poor	Moderate-poor	Moderate	Well	Well	ATCC
**p53 Mutation**	MT	MT	MT	MT	WT	[31]
**K-ras Mutation**	MT	MT	MT	WT	MT	[31]
**Colony-forming capacity**	+++	+++	+++	+	+	[32], UOD
**Spheroid-forming capacity**	+++	+	++	−	−	[32], UOD
**ALDH activity**	+++	++	+	+	+	[32], UOD
**Tumorigenic in mice**	+++	+++	Yes	+	Yes	[32], UOD
**E-cadherin Expression**	−	++	+	+++	+++	[33],[23] UOD
**Vimentin Expression**	+++	+++	++	+	−	[31]

ATCC: American Tissue Culture Collection; PDAC: Pancreatic Ductal Adenocarcinoma; UOD: unpublished own data; NE: not examined; −: none; +: weak; ++: median; +++: strong; MT: mutated; WT: wild type; Yes: present, but no quantification in relation to the other cell lines available.

**Figure 2:**
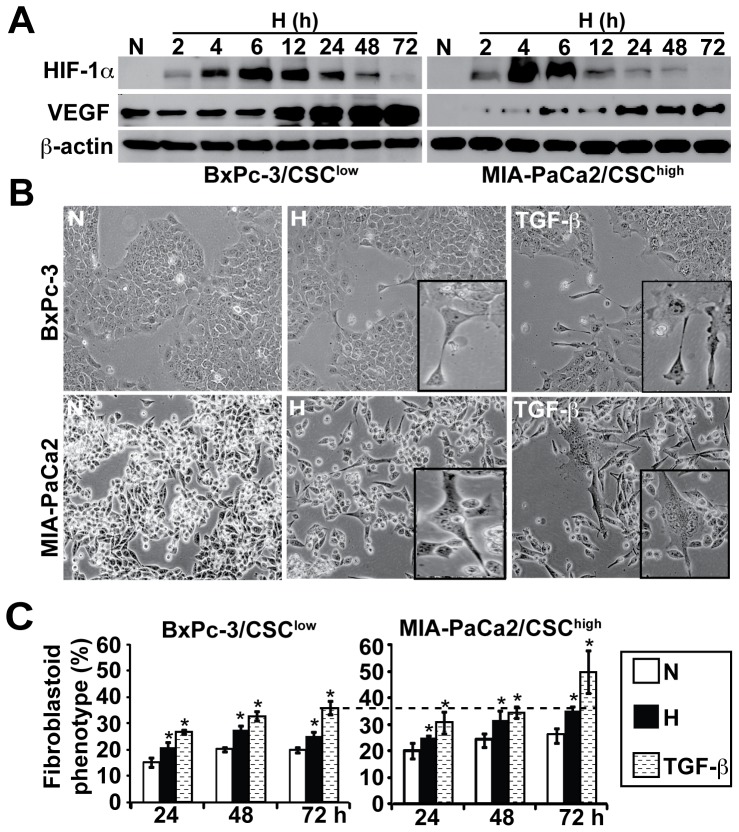
Response of pancreatic cancer cells to hypoxia in vitro. (**A**) For hypoxia induction *in vitro*, BxPc-3 and MIA-PaCa2 cells were incubated in an air-tight chamber in an atmosphere of 1% O_2_, 5% CO_2_ and 94% N_2_ for the time periods indicated. Controls were incubated under normoxic conditions for 48 h (N). Proteins were harvested and expression of HIF-1α and VEGF were examined by Western blot analysis. Protein extracts of cells cultured under normoxic conditions (N: 16% O_2_, 5% CO_2_, 79% N_2_) served as control for induction of hypoxia (H). Staining of Western blot membranes with β-actin served as control for equal conditions. (**B, C**) Morphological changes in cells exposed to hypoxia, normoxia, or TGF-β (5 ng/ml) were evaluated using a light microscope 72 h after incubation/treatment under 100*x* magnification (insets, 200*x*). The percentage of fibroblastoid spindle-shaped cells was counted at 24, 48 and 72 h.

1α and its target gene VEGF within 2 hours in both, CSC^low^ and CSC^high^ cells as examined by Western blot analysis. In contrast, cells cultured under normoxic conditions did not up-regulate these proteins (Fig. 2A, Data not shown). CSC^low^ cells had an expression peak between 6 and 12 hours, which gradually declined over time but was still visible at 72 h. In contrast, CSC^high^ cells had an earlier peak between 4 and 6 h, which was diminished to very low levels already at 24 hours and was undetectable at 72 h in both cell lines. In contrast, VEGF expression was steadily enhanced during a period of 72 h. In line with the observed hypoxia-related signaling the number of cells with a fibroblastoid-like phenotype increased within 72 h from 20 to 28% in CSC^low^ cells and from 28 to 33% in CSC^high^ cells (Fig. 2C). Induction of the percentage of spindle-shaped cells was even more pronounced and higher in CSC^high^ cells upon TGF-β, a well known-inducer of EMT, which was used as positive control. Similar morphological changes upon exposure to hypoxia were observed in Capan-1, Capan-2 and AsPC-1 cancer cells (Figure S1). These data suggest that *in vitro* induction of hypoxia induces EMT in both cell populations - more differentiated and CSC-like pancreatic cancer cells, but the effect is faster and stronger in CSC^high^ cells.

### Hypoxia up-regulates EMT-related protein expression

**Figure 3:**
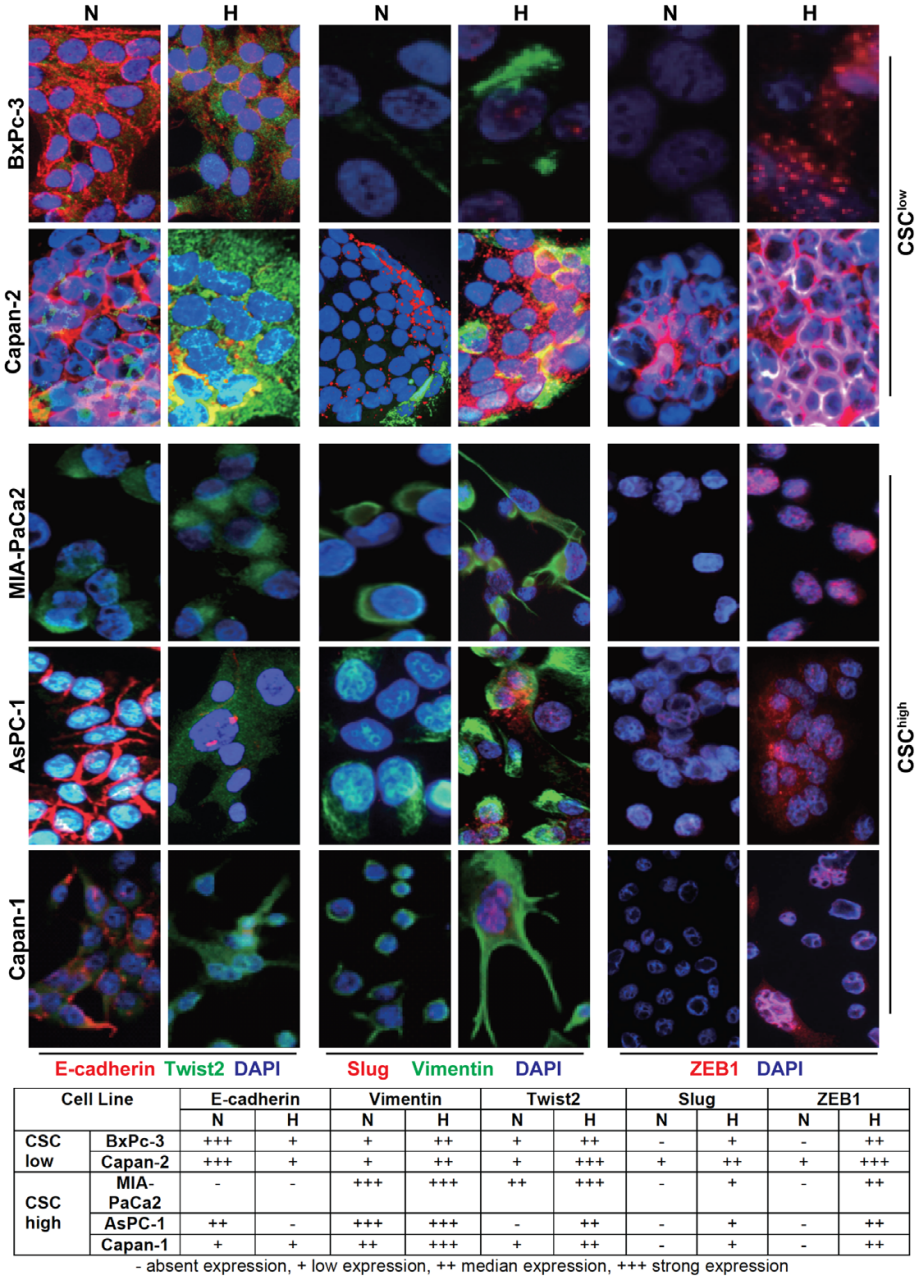
Hypoxia-induced expression of EMT-related proteins in vitro. BxPc-3 and Capan-2 CSC^low^ and MIA-PaCa2, AsPC-1 and Capan-1 CSC^high^ cells were exposed to normoxia or hypoxia for 48 h. Expression of EMT-related proteins was detected by double immunofluorescent staining followed by photographing the cells under 400*x* magnification. The size of pictures taken was further increased threefold in Photoshop. E-cadherin (red), Twist 2 (green), Slug (red), Vimentin (green), ZEB1 (red). Nuclei are stained with Dapi (blue). Changes in the levels of EMT-related proteins are shown below the photographs. No expression detectable (−), weak expression (+), median expression (++), strong expression (+++).

For further evaluation of cell-type specific effects of hypoxia-induced EMT we examined expression of proteins involved in EMT in the two CSC^low^ cell lines BxPc-3 and Capan-2 and in the three CSC^high^ cell lines MIA-PaCa2, AsPC-1 and Capan-1. After exposure to normoxia or hypoxia for 48 h we labeled the cells with specific antibodies and analyzed fluorescence by double immunofluorescence microscopy. While both CSC^low^ cell lines had strong basal expression of E-cadherin but low expression of Vimentin, as expected, hypoxia induces downregulation of E-cadherin and upregulation of Vimentin (Fig. 3). This was associated with upregulation of the EMT-markers Twist2, Slug and Zeb1. In contrast, CSC^high^ cells exhibited no (MIA-PaCa2) or a very low basal expression (AsPC-1, Capan-1) of E-cadherin but higher basal expression of Vimentin compared to CSC^low^ cells. Upon exposure to hypoxia a typical-EMT-like protein expression occurred similar as observed in CSC^low^ cells. Immunofluorescence data were confirmed by Western blot analysis (Data not shown). These data suggest that both, CSC^low^ and CSC^high^ cells exhibit the typical hypoxia-induced EMT-related gene expression but basal EMT-related protein expression under normoxic conditions is higher in CSC^high^ cells.

### Hypoxia stimulates migratory properties of pancreatic CSCs

**Figure 4:**
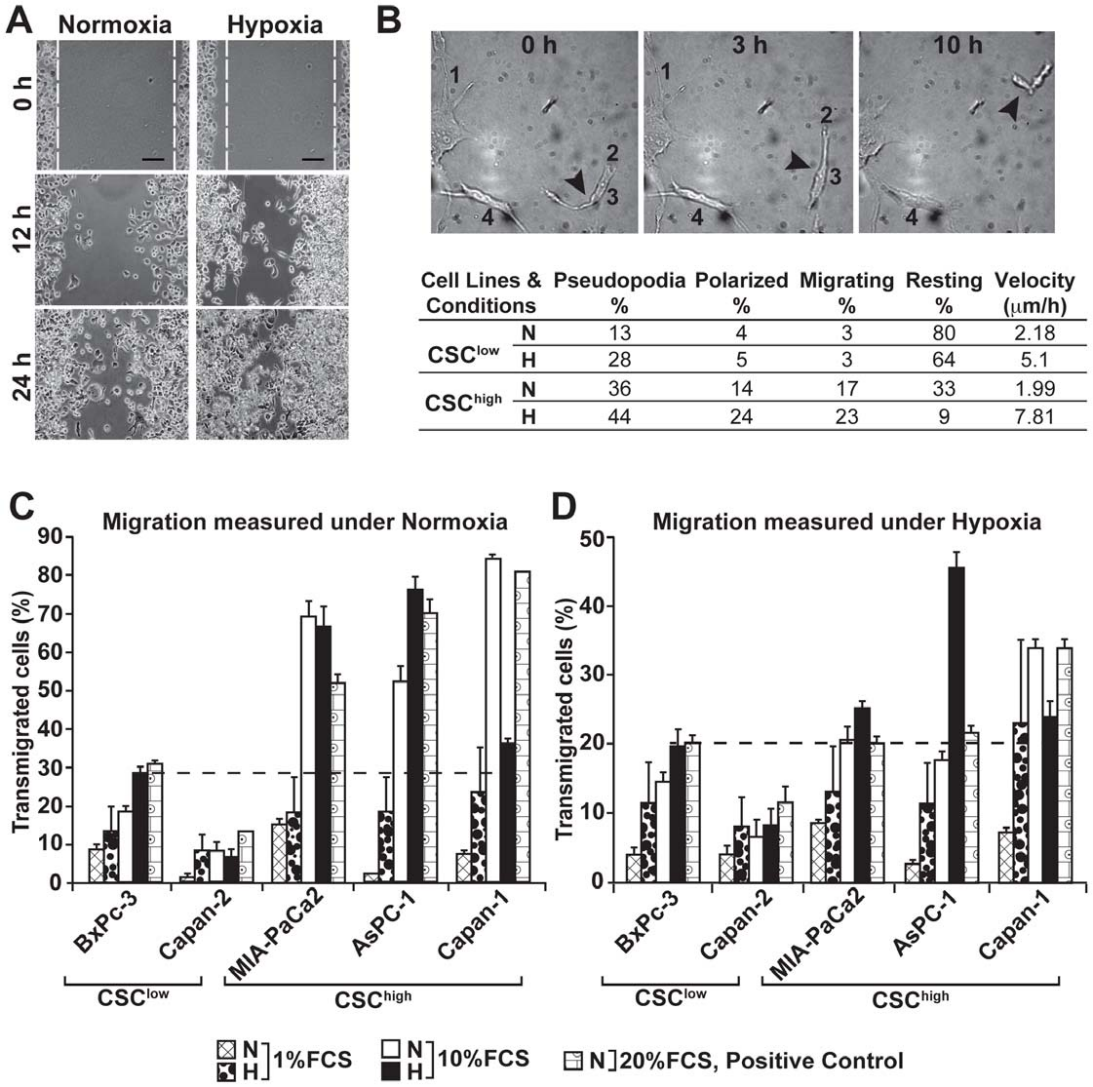
Effects of hypoxia on migration of pancreatic cancer cells. (**A**) Capan-1 cells were incubated under hypoxic (H) or normoxic (N) conditions for 24 h followed by scratching the confluent cell layer. Migration of cells into the wounded area was monitored during 24 h and photographs are shown at 0, 12 and 24 h after scratching. Bar: 100 μm. (**B**) BxPc-3 (CSC^low^) or AsPC-1 (CSC^high^) cells exposed to hypoxia (H) or normoxia (N) for 48 h were included in a three-dimensional (3D) collagen gel and locomotory activity was analyzed in a POCmini chamber by microscopy under 50*x* magnification as described in the Material & Methods section. Four types of cell locomotory activities were evaluated: active formation of pseudopodia (1), polarization as evident from cell elongation and bipolar shape formation (2), active migration to more than two cell diameters distance (arrow, 3) absence of a detectable cellular polarization and locomotion (4). Representative pictures are shown and time points of analysis are indicated. (**C**) BxPc-3, Capan-2, MIA-PaCa2, AsPC-1 and Capan-1 cells were pre-incubated under hypoxic (H) or normoxic (N) conditions. Transmigration was analyzed after additional 48 h of incubation under normoxic or hypoxic (**D**) conditions using a standard Transwell assay. Migration of cells towards medium with 1% or 10% FCS in the lower chamber was evaluated. Migration towards medium with 20% FCS served as positive control.

To investigate hypoxia-induced differences, we analyzed cancer cell migration in three different migration assays. First, we used a standard *in vitro* wounding assay. BxPc-3, Capan-2, MIA-PaCa2, AsPC-1 or Capan-1 cells were exposed to hypoxia or normoxia for 24 h followed by scratching of the confluent cell layer and further incubation at normoxic conditions. Images of migrating cells in the same area are shown 12 and 24 h after scratching. In all cell lines we found that the wounded region was closed faster by cells which had been exposed to hypoxia compared to cells exposed to normoxia. However, we could not detect any major differences in cell migration between CSC^high^ and CSC^low^ cell lines in this non-quantitative assay (Data not shown). Representative data for Capan-1 are shown (Fig. 4A). Therefore we used a more sensitive migration assay performed in 3D extracellular matrix. Migration of single cells was documented by time-lapse video microscopy. In line with the data obtained by Wound healing assay we found that 48 h exposure of CSC^high^ AsPC-1 or CSC^low^ BxPc-3 cells to hypoxia induced faster locomotion and a higher percentage of polarized, migrating and pseudopodia-forming cells than normoxia in both cell lines (Fig. 4B). Similar to our former results CSC^high^ cells migrated more rapidly than CSC^low^ cells. The mean velocity of AsPC-1 CSC^high^ cells exposed to hypoxia was 7.8 μm/h in contrast to 5.1 μm/h of CSC^low^ BxPc-3 cells. Also, the percentage of basal migrating cells as well as cells forming pseudopodia or exhibiting a polarized phenotype was higher in the CSC^high^ compared to CSC^low^ cells. These data were confirmed in a standard transwell assay. CSC^high^ MIA-PaCa2, AsPC-1, Capan-1 and CSC^low^ BxPc-3 and Capan-2 cells were pre-incubated under hypoxic conditions for 48 h in normal cell culture medium containing 10% FCS. Migration towards a gradient of 1%, 10% and 20% FCS under normoxia was measured by evaluating the number of transmigrated cells within 48 h (Fig. 4C). We found that pre-incubation of cells under hypoxic conditions increased the percentage of transmigrated cells in all cell lines. The most evident effects were obtained with cells moving towards a gradient of 1% FCS. At a gradient of 10% FCS basal migration of the CSC^high^ cells was already at the highest level as evident from the positive control with 20% FCS. Thus, hypoxia could not further induce basal migration of MIA-PaCa2 and Capan-1 cells towards a gradient of 10% FCS. Quite similar results were obtained when migration of hypoxia pre-treated cells was analyzed under hypoxic conditions (Fig. 4D). These results suggest that although hypoxia induces migration in more differentiated and CSC-like cells, basal and hypoxia-induced migratory potential is higher in CSC^high^ cells. Therefore hypoxia-induced EMT induces high migratory potential mainly in CSC-like cells, which may be responsible for invasion and metastasis.

### Co-expression of hypoxia- and EMT-markers is common in several tumor entities

**Figure 5:**
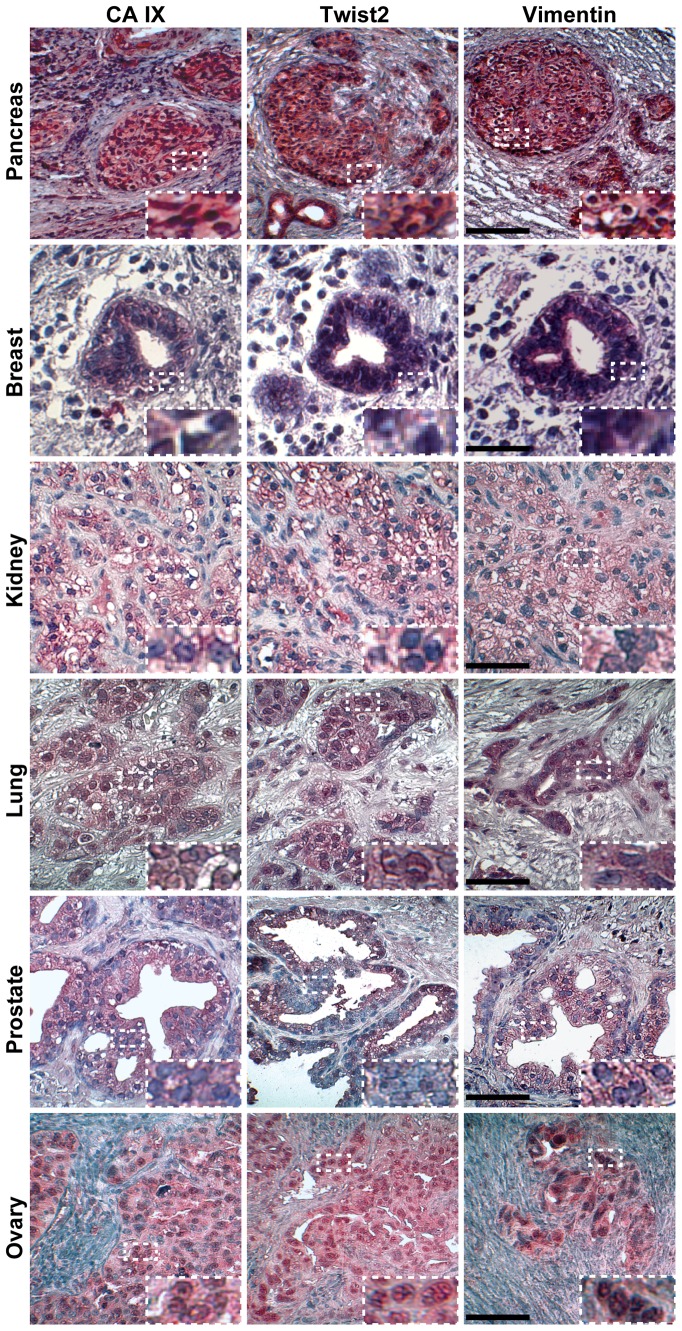
Co-expression of hypoxia and EMT markers in several cancer entities. The expression of carbonic anhydrase IX (CA IX), Twist2 and Vimentin was analyzed by regular immunohistochemistry in tumor tissues derived from patients with pancreatic (n = 5), breast (n = 11), kidney (n = 10), lung (n = 9), ovarian (n = 9) and prostate (n = 9) cancer. Positive cells (red) are red to dark-red. Bar: 100 μm. A threefold magnification (larger white squares) of a small area with positive-stained cells (smaller white squares) is shown within each picture.

Hypoxia-induced EMT was studied in paraffin-embedded patient-derived tissue samples of pancreatic, breast, kidney, lung, prostate and ovarian cancer. In all these tissues we identified expression of carbonic anhydrase IX (CA IX), a protein up-regulated by hypoxia [6], along with expression of Vimentin and the EMT-related transcription factor Twist2 (Fig. 5). In contrast, expression of CA IX, Vimentin and Twist2 was absent in non-malignant tissue as exemplified in normal lung tissue derived from the same patient from whom stainings of the corresponding malignant tissue are presented (Figure S2). These data demonstrate that hypoxia-driven EMT is a common feature of several tumor entities, which may be followed by enhanced migratory potential of the CSC population.

## Discussion

In the present study we evaluated the question whether hypoxia-induced EMT affects CSC-like cells of PDA only or also the more differentiated tumor cells. In patient tumor tissue of PDA we identified co-localization of the hypoxia marker HIF-1α and of the EMT marker Slug. Tissue regions of pancreatic cancer positive for the hypoxia marker HIF-2α were highly positive for the CSC marker CD133. *In vitro* both CSC^high^ and CSC^low^ pancreatic cancer cells responded to hypoxia by altering cell morphology from an epithelial to a more fibroblastoid or mesenchymal phenotype with a higher percentage in CSC^high^ cells. Morphological changes induced by hypoxia were associated with down-regulated E-cadherin expression and up-regulated expression of Vimentin, and EMT-related transcription factors Slug, Twist2 and Zeb1 in CSC^low^ cells. CSC^high^ cells had an protein expression signature of mesenchymal cells almost under normoxic conditions, as exemplified by low or absent E-cadherin levels along with high Vimentin expression, which was only marginally increased by hypoxia. This higher basal EMT status of CSC^high^ cells might be the reason for the observed increased migratory capacity in normoxic and hypoxic conditions. We assume that pancreatic stem-like tumor cells may have a survival advantage under unfavorable hypoxic conditions, since EMT has been demonstrated to contribute to drug resistance in pancreatic cancer [34]. In line with this assumption, MIA-PaCa2 and AsPC-1 with high basal EMT characteristics have been shown to be much more resistant toward Gemcitabine than BxPC-3 cells with low basal EMT features [34], which is also in agreement with the CSC-like phenotype of MIA-PaCa2 and AsPC-1.

Recent immunohistochemistry data confirm our results from double immunofluorescence staining of HIF-1α/Slug co-expression, since 87% of human PDA tissues out of 36 examined were found to express Snail and 50% of patients displayed positive expression of Slug, while Twist showed no or only weak expression [35]. However, Twist was strongly induced upon hypoxia in MIA-PaCa2, AsPC-1 and Capan-1 cells as detected by RT-PCR [35]. This matches to our findings of Twist2 expression in established and primary PDA cells, which was stronger upon induction of hypoxia *in vitro*. Another interesting report significantly strengthens these data since Twist RNA expression was found to be significantly higher in invasive PDAs compared to matched non-tumorous and IPMN samples [36].

The question is which molecular mechanisms are behind the enhanced EMT features and migratory capacities of CSC^high^ cells. In particular, EMT has been described to be dependent on NF-κB signaling in pancreatic cancer cells [37]. Since we recently identified enhanced NF-κB activity in CSC^high^ compared to CSC^low^ cells [32], NF-κB may be involved in mediating increased migratory properties of CSC^high^ cells. Another well known underlying reason for the very invasive growth pattern of e.g. MIA-PaCa2 cells seems to be that invasion is clearly dependent on CXCR4 [16]. Since pancreatic CSCs preferentially express CXCR4 receptor and metastasize using the CXCR4/SDF-1 axis [16] this may facilitate enhanced migratory potential. Furthermore, p53 is suggested to regulate EMT and stem cell properties and this may have contributed to the observed enhanced migratory potential of CSC^high^ cell lines, since MIA-PaCa2, AsPC-1, Capan-1 and BxPc-3, but not Capan-2 have mutated p53 [31]. Thus, loss of p53 correlates with an miR-200c-dependent increase in the expression of EMT, stemness markers and high tumor grade as described recently in a cohort of breast tumors [38]. Quite similarly, inhibition of p53 represses E-cadherin by promoter methylation as shown in ovarian tumor cells [39]. This observation is in agreement with the observed low E-cadherin expression in CSC^high^ cell lines with mutated p53.

We assume that the observed faster response of stem-like pancreatic tumor cells to hypoxia with upregulated EMT signaling and higher migratory potential may be even more pronounced in the *in vivo* tumor microenvironment. This may be due to the fact that in solid tumors repeated episodes of hypoxia followed by re-oxygenation is a common phenomenon. This so called “intermittent hypoxia” is described to regulate stem-like characteristics [40]. The occurrence of intermittent hypoxic episodes varies significantly in rapidly growing malignant tumors [41]. In phases of normoxia when EMT-inducing signals are removed CSC^low^ cells that have been induced to EMT may revert to the epithelial state similar by undergoing mesenchymal epithelial transition (MET) as has been reported to occur in some carcinoma cells [20], [42]. Additional new data provide insights into how dynamic interactions among epithelial, self-renewal, and mesenchymal gene programs determine the plasticity of CSC in switching between epithelial and mesenchymal states [43]. These findings suggest that CSC^low^ cells may differentiate to CSC-like cells upon induction of EMT by hypoxia and dedifferentiate upon withdrawal of hypoxia. In contrast, CSC^high^ cells with already basal enhanced EMT features may only partially undergo MET upon reversal of hypoxia. As a result the cells keep their high migratory potential in a normoxic tumor microenvironment and upon a new cycle of hypoxia they upregulate EMT signaling along with enhanced migratory activity faster. A higher basal EMT signaling and the ability to respond faster to EMT may provide a survival advantage to CSC^high^ cells. In consequence, this may lead to enrichment of CSCs during intermittent hypoxia in tumor progression and subsequently to enhanced invasion and metastasis.

In conclusion, we show that a hypoxic environment predominantly increases the migratory capacity of PDA cells with elevated stem cell characteristics. This is of important clinical relevance with respect to the pronounced hypoxic tumor-microenvironment of PDA [2]. Another contributing factor may be induction of hypoxia by anti-angiogenic medicaments like Sunitinib, Bevacizumab or Avastin and the observed pro-invasive adaption to such anti-angiogenic therapy [44], [45]. The results of the present study together with recent findings of other authors suggest the development of new treatment protocols to target tumor hypoxia.

## Supporting Information

Figure S1 **Morphological changes in pancreatic cancer cells exposed to hypoxia in vitro.** Morphological changes in established pancreatic cancer cell lines Capan-2, AsPC-1 and Capan-1 exposed to hypoxia (H) or normoxia (N) were evaluated using a light microscope under 100*x* magnification. Fibroblastoid spindle-shaped cells are indicated by arrows.
(EPS)

Figure S2 **Expression of hypoxia and EMT markers in normal lung tissue.** The expression of carbonic anhydrase IX (CA IX), Twist2 and Vimentin was analyzed by regular immunohistochemistry in normal lung tissue. Hematoxilin was used to counterstain the nuclei. Note the absence of the CA IX, Twist2 or Vimentin-positive cells (should appear in red).
(TIF)
