# Supplementary figures and images for: Hypoxia Induces EMT in Low and Highly Aggressive Pancreatic Tumor Cells but Only Cells with Cancer Stem Cell Characteristics Acquire Pronounced Migratory Potential
Source: PLoS One. 2012 Sep 26;7(9):e46391. doi: 10.1371/journal.pone.0046391 (PMC3458836; doi:10.1371/journal.pone.0046391)

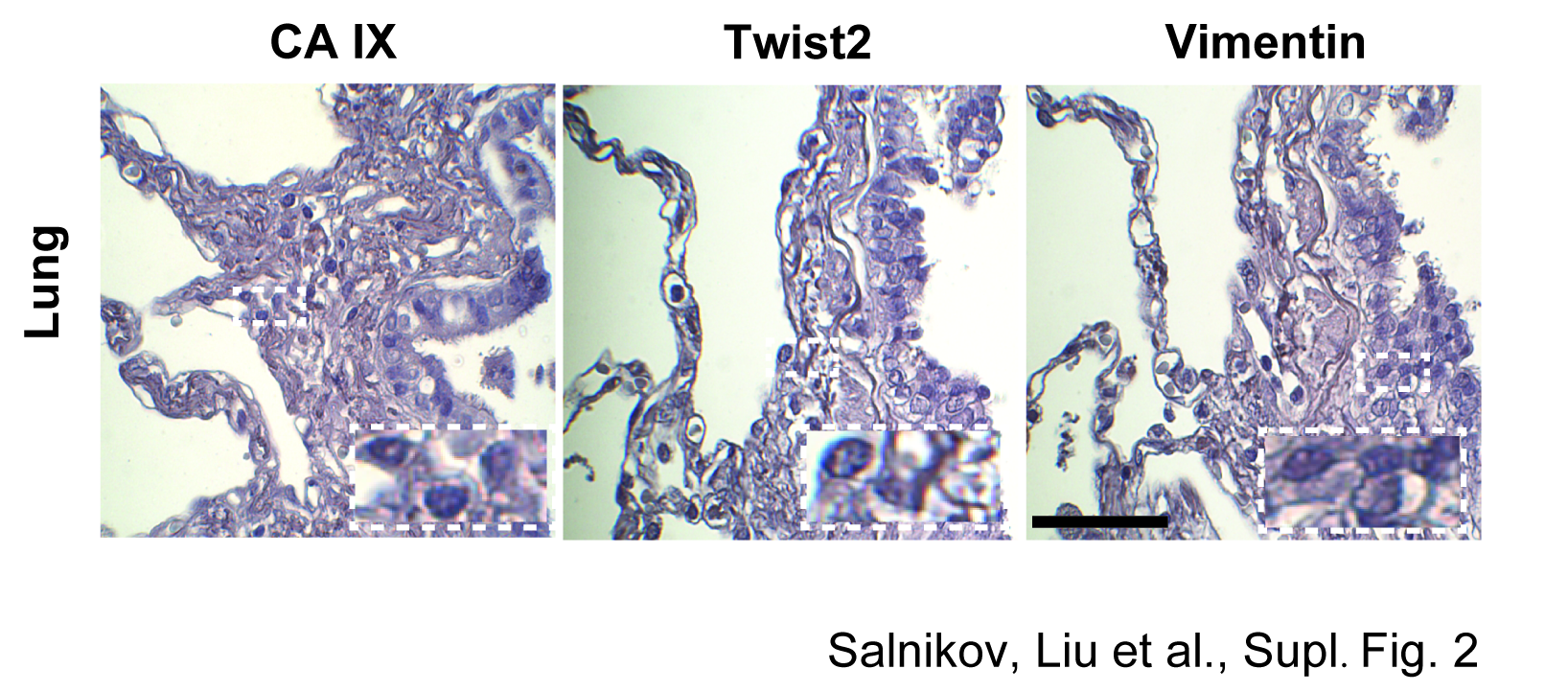

Supplement: Figure S2 — Expression of hypoxia and EMT markers in normal lung tissue. The expression of carbonic anhydrase IX (CA IX), Twist2 and Vimentin was analyzed by regular immunohistochemistry in normal lung tissue. Hematoxilin was used to counterstain the nuclei. Note the absence of the CA IX, Twist2 or Vimentin-positive cells (should appear in red). (TIF) [file pone.0046391.s002.tif]
